# Postoperative circulating tumor DNA testing based on tumor naïve strategy after liver metastasis surgery in colorectal cancer patients

**DOI:** 10.3389/fonc.2023.1153685

**Published:** 2023-04-28

**Authors:** Huiqin Jiang, Fei Huang, Yihui Yang, Xinning Chen, Minna Shen, Chunyan Zhang, Baishen Pan, Beili Wang, Wei Guo

**Affiliations:** ^1^ Department of Laboratory Medicine, Zhongshan Hospital, Fudan University, Shanghai, China; ^2^ Department of Laboratory Medicine, Xiamen Branch, Zhongshan Hospital, Fudan University, Xiamen, China; ^3^ Department of Laboratory Medicine, Wusong Branch, Zhongshan Hospital, Fudan University, Shanghai, China; ^4^ Branch of National Clinical Research Center for Laboratory Medicine, Shanghai, China; ^5^ Cancer Center, Zhongshan Hospital, Fudan University, Shanghai, China

**Keywords:** minimal residual disease (MRD), circulating tumor DNA (ctDNA), colorectal cancer liver metastases (CRLM), next-generation sequencing – NGS, recurrence

## Abstract

**Objective:**

There is still a lack of highly sensitive methods for monitoring recurrence of colorectal cancer patients after liver metastasis surgery. The aim of this study was to evaluate the prognostic value of tumor-naive ctDNA detection after resection of colorectal liver metastases (CRLM).

**Methods:**

Patients with resectable CRLM were prospectively enrolled. Based on the tumor-naive strategy, NGS panels containing 15 colorectal cancer hotspot mutated genes were used to detect ctDNA 3-6 weeks after surgery.

**Results:**

A total of 67 patients were included in the study, and the positive rate of postoperative ctDNA was 77.6% (52/67). Patients with positive ctDNA had a significantly higher risk of recurrence after surgery (HR 3.596, 95% CI 1.479 to 8.744, P = 0.005), and a higher proportion relapsed within 3 months after surgery (46.7% *vs* 3.8%). The C-index of postoperative ctDNA in predicting recurrence was higher than that of CRS and postoperative CEA. The nomogram combining CRS and postoperative ctDNA can improve the accuracy of recurrence prediction.

**Conclusion:**

Tumor-naive ctDNA detection can detect molecular residual lesions in patients with colorectal cancer after liver metastasis, and its prognostic value is superior to conventional clinical factors.

## 1 Introduction

Colorectal cancer (CRC) is one of the most common malignancies, accounting for 10% of all cancers, and is the second leading cause of cancer death, with approximately 1.9 million new cases and 90,000 deaths worldwide in 2020 (1). The liver is one of the most common metastatic sites of CRC, with an incidence as high as 25% – 30% (2). With the continuous improvement of treatment methods, including surgery of liver metastasis and novel anticancer drugs, the prognosis of CRC patients with liver metastases (CRLM) has been significantly improved (3). However, it has been shown that radical resection of liver metastases improves survival by 40% in patients with CRLM (4), and approximately 50% relapse postresection (5). Repeated surgical treatment has been proven effective for liver and lung recurrence after CRLM resection when the recurrent lesion remains curatively resectable (6). Therefore, early detection of postoperative recurrence is the key to improving the prognosis of these patients.

The traditional means of postoperative follow-up mainly include imaging (B ultrasound, CT, magnetic resonance imaging, etc.) and tumor markers (CEA). However, studies have shown that the sensitivity of CEA in detecting recurrence is limited, ranging from only 68% to 82% (7). Imaging can only detect overt lesions and has shown limited sensitivity in detecting recurrent metastatic disease (8). Assessing the prognosis of patients with solid tumors based on postoperative pathological parameters is the most commonly used strategy. The clinical risk score (CRS) scoring system, established in 1999, is a recognized prognostic indicator after resectioning CRC liver metastases and has also been included in clinical guidelines. CRS score consists of five parameters: (1) positive lymph node metastasis from the primary tumor, (2) >1 liver metastasis, (3) largest diameter of liver metastasis >50mm, (4) preoperative CEA level >200ng/ml, and (5) disease-free interval <12 months between resection of the primary tumor and diagnosis of liver metastasis (9). Each item is 1 point, 0 to 2 points for low CRS scores, and 3 to 5 points for high CRS scores. The higher the CRS score, the greater the risk of postoperative recurrence and the more beneficial perioperative chemotherapy. However, this predictive model based on preoperative clinical and postoperative pathological parameters remains somewhat imprecise, and we need a more precise way that directly reflects postoperative residual disease.

Since circulating tumor DNA (ctDNA) has a short half-life (ranging from minutes to a few hours) and allows for a more accurate, real-time, and dynamic measure of tumor burden, it has emerged as an ideal biomarker (10). Previous studies in various solid tumors have shown that postoperative ctDNA detection of molecular residual disease (MRD) can better identify patients with early recurrence (11–13). Several studies of early colorectal cancer have yielded similar results (13–18). However, there are still many controversies about ctDNA-based MRD detection strategies, including panel selection, the cutoff value of MRD positivity, and the timing of ctDNA testing. In previous studies, the tumor-informed strategy was mainly used to detect MRD; that is, tumor-specific genetic alterations were identified by whole-exome sequencing or targeted sequencing of the primary tumor (e.g., SignateraTM, SafeSeqS) from each patient to track them in ctDNA samples (13, 19). However, this strategy increases turnaround time (TAT) and has a certain failure rate. It has been shown that the QC failure rate for sequencing is as high as 16.9% due to poor-quality tumor tissue samples (20). In contrast, the tumor-naïve strategy performed without prior knowledge of the patient’s tumor mutational profile using a fixed panel, which has several advantages, including fast TAT, logistical simplicity, and low cost.

In this study, we investigate the clinical validity of postoperative ctDNA testing by using a tumor-naive NGS panel containing colorectal cancer hotspot mutations.

## 2 Methods

### 2.1 Patient enrollment and sample collection

Patients with resectable CRC liver metastases (CRLM) were recruited for this prospective study. The main inclusion criteria included: pathologically confirmed colorectal cancer, primary lesions resected, and underwent liver metastasectomy with curative intent. The main exclusion criteria included: extrahepatic metastasis and a history of other cancers. All patients underwent standard preoperative staging investigations to assess liver lesions and the presence of metastases at other sites, including liver MRI and chest CT, or PET-CT. To assess the resectability of liver lesions, all patients underwent a multidisciplinary discussion (MDT) with our hospital’s team of experts. The MDT team consisted of colorectal surgeons, liver surgeons, imaging physicians, radiotherapy physicians, medical oncologists, and pathologists. Blood samples for ctDNA analysis were collected 3-6 weeks after liver resection before commencing chemotherapy. At least 10 mL of blood was drawn into EDTA tubes, centrifuged twice at 3000rpm and 14000rpm, and plasma was then stored at −80°CC for ctDNA analysis. The study complied with the ethical standards of the Declaration of Helsinki and was reviewed and approved by the institutional ethics committee (Zhongshan Hospital Fudan University; B2018-099). Written informed consent was obtained from all participants.

### 2.2 ctDNA analysis

A tumor-naive strategy was used to detect postoperative ctDNA (Oncomine ™ Colon cfDNA), covering 14 colorectal cancer hotspot genes: AKT1, APC, BRAF, CTNB1, EGFR, ERBB2, FBXW7, GNAS, KRAS, MAP2K1, NRAS, PIK3CA, SMAD4, and TP53, which were detected at Illumina MiSeq (Illumina, USA) next-generation sequencing platform. Using tag sequencing technology, a limit of detection (LOD) as low as 0.1% can be achieved. Plasma samples with at least one mutation detected above a predefined confidence threshold were deemed ctDNA positive.

### 2.3 Clinicopathological data and follow-up

Clinicopathological characteristics were collected based on medical history records, including age, gender, primary tumor location, time to metastases, size and number of metastases, serum CEA levels, and clinical risk score (CRS). All patients received standard-of-care postoperative treatment and surveillance, according to the investigator’s choice, per protocol follow-up after liver resection included clinical review, CEA evaluation, and imaging exam every three months.

### 2.4 Statistical analyses

The primary objective was to measure the recurrence-free survival (RFS) from the time of surgery to the first radiologic evidence of disease progression or a CRC-caused death. Patients were censored by the end of follow-up or by a non-CRC- caused death. Survival analyses were performed using the Kaplan-Meier method. The performance of ctDNA as a marker of RFS outcome was evaluated using the concordance index (C-index). C-index of 0.5–0.7, 0.7–0.85, and 0.85–0.95 were defined as low, middle, and high credibility, respectively. Group comparisons were performed using chi-square tests or Fisher exact test, as appropriate. SPSS23.0 (SPSS Inc., Chicago, IL) and R (R version 3.2.1, http://www.r-project.org) were used for statistical analysis. All P values were based on two-sided testing, and differences were considered significant at P ≤ 0.05.

## 3 Results

### 3.1 Associations between postoperative ctDNA status and clinicopathologic factors

A total of 67 patients were included in this study, and the median time from the date of liver surgery to postoperative blood collection was 28 days (inter-quartile range (IQR), 23.5 to 34 days). Of these, 22.4% (15/67) of patients were positive for ctDNA after surgery, and the remaining 77.6% (52/67) were negative. The differences in clinicopathologic characteristics between the postoperative ctDNA positive and negative groups were analyzed. The results showed that CRS score, primary tumor location, whether synchronous liver metastasis, number of liver metastases, the maximum diameter of liver metastases, RAS/RAF status, and preoperative and postoperative CEA levels were not significantly correlated with postoperative ctDNA status (**Table 1**).

**Table 1:** Relationship between clinic-pathological variables and postoperative ctDNA status.

	Postoperative ctDNA negative	Postoperative ctDNA positive	*P* value
CRS			0.281
Low (score 0-2)	23 (44.2%)	9 (60.0%)	
High (score 3-5)	29 (55.8%)	6 (40.0%)	
Location of primary tumor			0.344
Left colon	17 (32.7%)	8 (53.3%)	
Right colon	14 (26.9%)	3 (20.0%)	
Rectum	21 (40.4%)	4 (26.7%)	
Synchronous liver metastases			0.742
Yes	37 (71.2%)	12 (80.0%)	
No	15 (28.8%)	3 (20.0%)	
Number of liver metastasis			1.000
Single	37 (71.2%)	11 (73.3%)	
Multiple	15 (28.8%)	4 (26.7%)	
Diameter of largest liver metastasis			1.000
≥5cm	9 (17.3%)	2 (13.3%)	
<5cm	43 (82.7%)	13 (86.7%)	
KRAS/NRAS/BRAF			0.321
WT	16 (30.8%)	2 (13.3%)	
MT	36 (69.2%)	13 (86.7%)	
Preoperative CEA			0.568
≥200ng/mL	4 (7.7%)	0 (0.0%)	
<200ng/mL	48 (92.3%)	15 (100.0%)	
Postoperative CEA			0.542
≥5ng/mL	16 (30.8%)	6 (40.0%)	
<5ng/mL	36 (69.2%)	9 (60.0%)	

CRS, clinical risk score; WT, wild type; MT, mutant type; CEA, carcinoembryonic antigen.

### 3.2 Postoperative ctDNA status predicts early recurrence

At the time of data cutoff, 41 of 67 patients had a recurrence, of which 9 had a recurrence within three months after surgery, with a median follow-up time of 9.67 months. Early recurrence was only associated with postoperative ctDNA status but not with age, gender, primary tumor location, preoperative and postoperative CEA, and CRS scores. Of the postoperative ctDNA-positive patients, 46.7% (7/15) developed recurrence within three months after surgery; however, only 3.8% (2/52) developed early recurrence in the postoperative ctDNA-negative group (**Table 2**).

**Table 2:** Analysis of factors associated with short-term recurrence.

	Total	Recurrence within 3 months	No recurrence within 3 months	*P* value
All patients, n	67	9	58	
Age				0.714
≥ 65y	20	2 (22.7%)	18 (31.0%)	
<65y	47	7 (77.8%)	40 (69.0%)	
Gender				0.721
Male	38	6 (66.7%)	32 (55.2%)	
Female	29	3 (33.3%)	26 (44.8%)	
Location of primary tumor				0.449
Left colon	25	5 (55.6%)	20 (34.5%)	
Right colon	17	2 (22.2%)	15 (25.9%)	
Rectum	25	2 (22.2%)	23 (39.7%)	
LN from primary tumor				0.671
Positive	52	8 (88.9%)	44 (75.9%)	
Negative	15	1 (11.1%)	14 (24.1%)	
Time interval from diagnosis of primary tumor to liver metastases				0.186
≥12 months	14	0 (0.0%)	14 (24.1%)	
<12 months	53	9 (100.0%)	44 (75.9%)	
Synchronous liver metastases				0.426
Yes	49	8 (88.9%)	41 (70.7%)	
No	18	1 (11.1%)	17 (29.3%)	
Number of liver metastasis				0.706
Single	48	6 (66.7%)	42 (72.4%)	
Multiple	19	3 (33.3%)	16 (27.6%)	
Diameter of largest liver metastasis				0.336
≥5cm	11	0 (0.0%)	11 (19.0%)	
<5cm	56	9 (100.0%)	47 (81.0%)	
KRAS/NRAS/BRAF				0.426
WT	18	1 (11.1%)	17 (29.3%)	
MT	49	8 (88.9%)	41 (70.7%)	
Preoperative CEA				1.000
≥200ng/mL	4	0 (0.0%)	4 (6.9%)	
<200ng/mL	63	9 (100.0%)	54 (93.1%)	
Postoperative CEA				0.461
≥5ng/mL	22	4 (44.4%)	18 (31.0%)	
<5ng/mL	45	5 (55.6%)	40 (69.0%)	
CRS				1.000
Score 0-2	32	4 (44.4%)	28 (48.3%)	
Score 3-5	35	5 (55.6%)	30 (51.7%)	
Postoperative ctDNA (day 30)				**0.000**
Positive	15	7 (77.8%)	8 (13.8%)	
Negative	52	2 (22.2%)	50 (86.2%)	

LN, lymph node; WT, wild type; MT, mutant type; CEA, carcinoembryonic antigen; CRS, clinical risk score. Bold values indicates that the P value have statistically significant differences.

**Figure 1:**
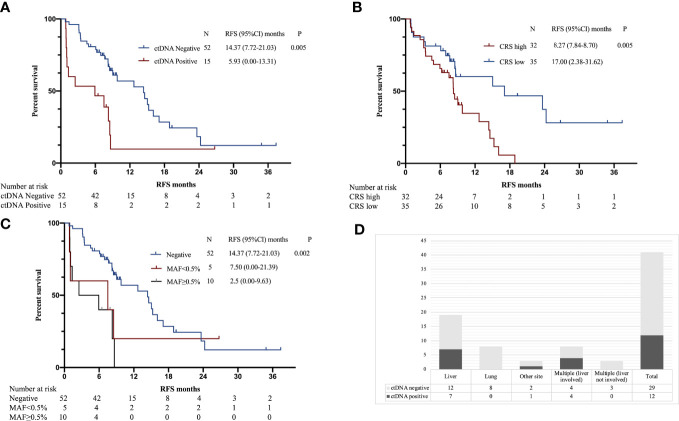
**(A)** Kaplan–Meier estimates of recurrence-free survival (RFS) for postoperative ctDNA. **(B)** Kaplan–Meier estimates of RFS for CRS. **(C)** Kaplan–Meier estimates of RFS for postoperative ctDNA MAF. **(D)** Postoperative ctDNA positivity according to the site of recurrence.

Univariate survival analysis was performed to assess the ability of postoperative ctDNA status to predict recurrence compared with other clinicopathological variables. The results showed that RFS was significantly associated with postoperative ctDNA status and CRS score (**Table 3**). The postoperative ctDNA-positive group had a considerably shorter RFS than the ctDNA-negative group (5.93 *vs*. 14.30, P = 0.005, HR 3.596, 95% CI 1.479 to 8.744); the high CRS group had a significantly shorter RFS than the low CRS group (8.27 *vs*. 17.00, P = 0.005, HR 2.517, 95% CI 1.317 to 4.810) (**Figures 1A, B**).

**Table 3:** Recurrence-free survival analysis by clinic-pathological variables.

	Median RFS (95% CI) months	*P* value
Age		0.597
<65y	9.030 (7.412-10.648)	
≥ 65y	16.000 (0.000-33.412)	
Gender		0.165
Female	8.200 (5.888-10.512)	
Male	14.500 (7.177-21.823)	
Location of primary tumor		0.335
Left colon	12.700 (5.890-19.510)	
Right colon	8.230 (4.845-11.615)	
Rectum	15.000 (5.461-24.539)	
LN from primary tumor		0.063
Positive	8.500 (7.481-9.519)	
Negative	18.800 (6.277-31.323)	
Time interval from diagnosis of primary tumor to liver metastases		0.061
≥12 months	15.000 (2.549-27.451)	
<12 months	8.630 (7.622-9.638)	
Synchronous liver metastases		0.227
Yes	9.030 (7.401-10.659)	
No	14.300 (3.893-24.707)	
Number of liver metastasis		0.491
Single	9.800 (3.827-15.773)	
Multiple	7.630 (0.071-15.189)	
Diameter of largest liver metastasis		0.146
≥5cm	7.500 (1.328-13.672)	
<5cm	9.800 (5.147-14.453)	
KRAS/NRAS/BRAF		0.377
WT	15.200 (undetermined)	
MT	8.500 (6.446-10.554)	
Preoperative CEA		0.344
≥200ng/mL	6.230 (0.781-11.679)	
<200ng/mL	9.800 (4.611-14.989)	
Postoperative CEA		0.371
<5ng/mL	9.800 (4.330-15.270)	
≥5ng/mL	8.470 (5.964-10.976)	
CRS		**0.005**
Low risk (score 0-2)	17.000 (2.377-31.623)	
High risk (score 3-5)	8.270 (7.844-8.696)	
Postoperative ctDNA (day 30)		**0.005**
Negative	14.300 (7.743-20.857)	
Positive	5.930 (0.000-13.309)	

LN, lymph node; WT, wild type; MT, mutant type; CEA, carcinoembryonic antigen; CRS, clinical risk score. Bold values indicates that the P value have statistically significant differences.

Out of 67 patients, 28 (42%) received a combination of targeted therapy (cetuximab or bevacizumab) and fluoropyrimidine-containing doublet chemotherapy (FOLFOX/FOLFIRI), while 39 (58%) received only fluoropyrimidine-containing doublet chemotherapy post-surgery. The duration of chemotherapy did not exceed six months for any patient. Statistical analysis revealed no significant difference in progression-free survival (PFS) between patients who underwent targeted combination chemotherapy and those who only received chemotherapy (P = 0.546). Additionally, our study found that the postoperative ctDNA status was significantly associated with PFS in both the targeted combined chemotherapy and chemotherapy groups (P = 0.002, P = 0.050) (**Figure S1**). Therefore, the results suggest that the predictive ability of postoperative ctDNA status for recurrence is not influenced by the type of postoperative treatment regimen administered.

### 3.3 Development of a predictive nomogram

**Figure 2:**
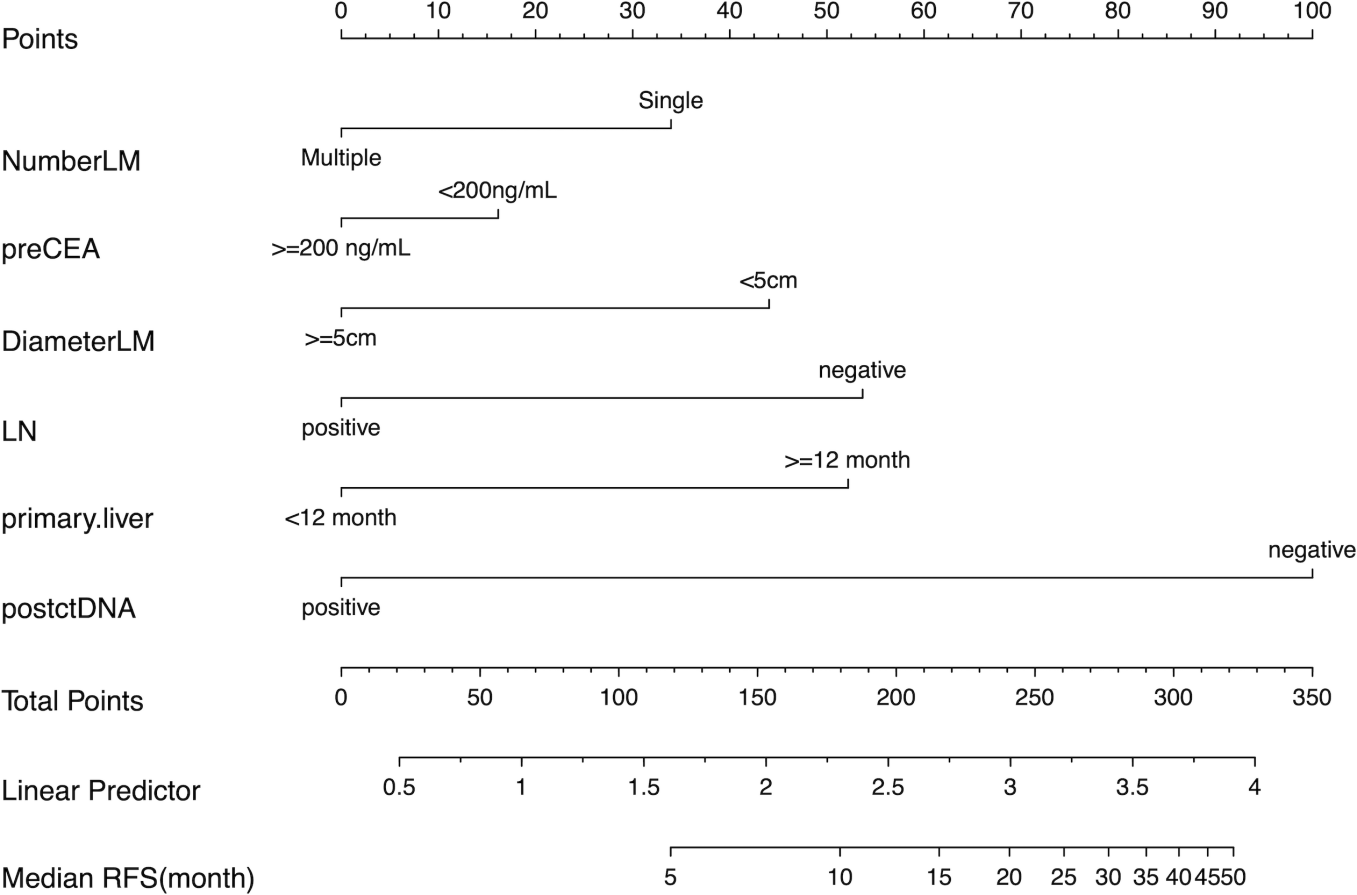
The Nomogram for predicting recurrence-free survival (RFS) in patients undergoing curative resection of colorectal cancer liver metastases. Number of liver metastasis (NumberLM), Preoperative CEA level (preCEA), Largest diameter of liver metastasis (DiameterLM), lymph node metastasis from the primary tumor, Postoperative ctDNA (postctDNA).

The previous survival analysis showed that CRS and postoperative ctDNA status were prognostic factors, so we developed a nomogram recurrence prediction model. As mentioned earlier, the CRS score contained five clinical variables, namely, preoperative CEA level, number of liver metastases, the maximum diameter of liver metastases, lymph node metastasis status of the primary tumor, and the time interval between the primary tumor and liver metastases, combined with postoperative ctDNA, a total of six variables were included in the nomogram model (**Figure 2**).

Subsequently, The C-index was used to evaluate the discrimination power of postoperative CEA, CRS, postoperative ctDNA, and the nomogram. The accuracy of postoperative ctDNA in predicting recurrence was higher than that of CRS and postoperative CEA (C-index 0.619 *vs*. 0.583 *vs*. 0.542), and the nomogram model had the highest C-index of 0.702, indicating that the multi-parameter model combining CRS and postoperative ctDNA can improve the accuracy of recurrence prediction. (**Table 4**)

**Table 4:** C-index for the nomogram, postoperative CEA, CRS and postoperative ctDNA.

Variable	C-Index	95CI%
Postoperative CEA	0.542	0.454-0.630
CRS	0.583	0.491-0.675
Postoperative ctDNA	0.619	0.537-0.701
Nomogram	0.702	0.604-0.800

CEA, carcinoembryonic antigen; CRS, clinical risk score.

### 3.4 ctDNA MAF and RFS

Of the 15 patients with positive postoperative ctDNA, 10 had MAF ≥ 0.5%, and 5 had MAF < 0.5%, the median MAF was 0.93% (IQR 0.35% to 2.09%). Patients in the MAF ≥ 0.5% group had significantly shorter RFS than those in the MAF < 0.5% group and those in the ctDNA-negative group (2.5 *vs*. 7.5 *vs*. 14.37, P = 0.002) (**Figure 1C**).

### 3.5 Postoperative ctDNA Status and site of recurrence

Previous studies have shown that metastatic sites are associated with detection rates of ctDNA (21). Therefore, we analyzed the relationship between the site of recurrence and postoperative ctDNA. Among the 41 patients with recurrence, the detection rate of ctDNA was 29.3% (12/41). Postoperative ctDNA was detected in 37% (7/19) of patients with liver recurrence, 36% (4/11) patients with multiple sites of recurrence, 0% (0/8) of patients with lung recurrences and 33% (1/3) of patients with recurrence to other sites (peritoneal or lymph nodes) (**Figure 1D**).

## 4 Discussion

This study used a tumor-naïve strategy to detect postoperative ctDNA status in mCRC who underwent resection of liver metastases. The results showed that the positive rate was 22.4% (15/67). Patients with positive ctDNA had significantly shorter RFS (5.93 *vs*. 14.30, P = 0.005, HR 3.596, 95% CI 1.479 to 8.744) and a considerably higher proportion of recurrence within three months after surgery (46.7% *vs*. 3.8%) compared with negative patients. Two previous studies based on tumor-informed strategies showed that postoperative ctDNA positivity in patients undergoing curative resection of CRLM was 24% and 54.4%, and positive ctDNA both indicated shorter RFS (HR 6.3, 95% CI 2.58 – 15.2, P < 0.001; HR 5.78, 95% CI 3.34 – 10.0, P 0.001) (20, 22). Compared with the previous two studies using a tumor-informed strategy, our study using a tumor-naïve strategy showed slightly lower ctDNA positivity as well as a lower hazard ratio for recurrence. In this study, the sensitivity of the tumor-naïve strategy for detecting postoperative MRD was 37%. Previous studies on early-stage colorectal cancer reported a sensitivity of approximately 40%-50% for MRD detection using a tumor-informed strategy one month after surgery^18,19.^ However, these studies focused on patients with stage II/III colorectal cancer, with a median follow-up time of 1-2 years and a postoperative recurrence rate of 15% to 18%. In contrast, our study focused on colorectal cancer patients with liver metastasis, with a median follow-up time of 9.67 months and a significantly higher postoperative recurrence rate of 61%. The sensitivity of ctDNA detection is influenced by disease stage and tumor burden, with a higher ctDNA positivity rate in patients with advanced disease stages or higher tumor burdens (23). Therefore, the relatively high sensitivity of MRD detection in our study is largely attributed to the advanced stage of disease in the study population.

It is generally accepted that the sensitivity of the tumor-naïve strategy is lower than that of the tumor-informed strategy. Combining the detection of multiple types of markers, such as methylation markers, can improve the sensitivity of MRD detection using the tumor-naïve strategy (16). For example, a study in colorectal cancer found that integrating methylation signatures increased sensitivity by 25%–36% versus genomic alterations alone (16). Therefore, we believe that the future trend in applying the tumor-naïve strategy to MRD detection is to combine methylation and mutation markers. It is worth noting that in our study, we only analyzed MRD at a single “Landmark” time point and did not perform longitudinal monitoring of ctDNA. However, MRD results at the “Landmark” time point (usually about one month after curative treatment) have important clinical significance for predicting patient prognosis and making treatment decisions. Studies have shown that continuous monitoring can improve the sensitivity of recurrence monitoring (18). Nevertheless, challenges in MRD detection remain, such as the need for a large amount of blood collection and high costs. Therefore, the clinical utility of longitudinal continuous monitoring needs to be further verified. Nevertheless, our study is able to show that tumor-naïve ctDNA assay is effective in identifying patients with relapse. In addition, since the tumor-naïve strategy has a fixed panel and short TAT, it has certain advantages in clinical applications in the future.

Currently, most studies categorized ctDNA results as either positive or negative, and few investigated the relationship between ctDNA MAF and recurrence risk. This study found that higher MAF was associated with shorter RFS (mRFS, 2.5, 7.5, and 14.37 months for MAF of ≥ 0.5%, < 0.5%, and 0%, P = 0.002). In other words, Recurrence risk increased with increasing ctDNA MAF. Then, we need to consider whether ctDNA predicts recurrence no longer accurately when MAF is reduced to a certain extent. This question also determines what limit we pursue the sensitivity of ctDNA detection. A study in early colorectal cancer showed the HR for recurrence was only 1.2 for postoperative ctDNA MAF of 0.1% (24). In addition, as the detection sensitivity increases, the specificity decreases, especially the interference of clonal hematopoiesis. Therefore, in real-world clinical applications, we need to balance multiple factors such as sensitivity, specificity, TAT, and cost rather than just pursuing the limit of a single parameter.

Previous studies have found that ctDNA detection can vary by the site of metastases. Kagawa et al. observed that CRC patients with liver metastases were associated with increased ctDNA detection rates compared to patients with lung and peritoneal metastases (21). Therefore, we further analyzed the relationship between sites of recurrence and postoperative ctDNA positivity. We also found that the site of recurrence affected the ability to detect ctDNA prior to radiological diagnosis of recurrence. The results showed that the rate of positive ctDNA was higher in patients with liver metastasis than those with other sites of metastasis (such as lung and peritoneal) after CRLM surgery. This difference may be associated with physiological barriers or tumor burden, but the specific mechanisms remain to be explored.

It is important to emphasize that ctDNA outperforms conventional clinical parameters (CEA, CRS) in predicting cancer relapse in patients with resected CRLM. The accuracy of postoperative ctDNA in predicting recurrence was higher than that of CRS and postoperative CEA (C-index 0.619 *vs*. 0.583 *vs*. 0.542). In this study, we combined postoperative ctDNA status with traditional prognostic markers to construct a nomogram predicting recurrence after CRLM surgery to assist in distinguishing patients at high risk of recurrence who may require more aggressive treatment as well as closer follow-up strategies. The nomogram provides better predictive accuracy for postoperative recurrence than traditional prognostic markers (CEA, CRS) in CRLM patients.

There are several limitations to our study. These include a small sample size, a lack of a validation cohort, and a relatively short follow-up period. In addition, this study did not test the preoperative ctDNA level and it is unclear what percentage of patients had negative preoperative ctDNA. Theoretically, patients with negative preoperative ctDNA may not have their disease recurrence effectively reflected by postoperative ctDNA status. However, the present study demonstrated the potential use of tumor-naïve ctDNA analysis as a prognostic tool. The results also revealed the correlation between preoperative ctDNA detection and the site of recurrence, as well as the impact of ctDNA MAF on RFS. All these factors are essential to consider in future MRD testing.

## 5 Conclusion

In summary, we have confirmed the prognostic significance of detecting ctDNA by tumor-naïve strategy in patients undergoing resection for CRLM. Nomogram based on postoperative ctDNA and CRS might be promising biomarkers in future trials to select high-risk CRLM patients for personalized therapy.

## Data availability statement

The raw data supporting the conclusions of this article will be made available by the authors, without undue reservation.

## Ethics statement

The studies involving human participants were reviewed and approved by Zhongshan Hospital Fudan University(B2018-099). The patients/participants provided their written informed consent to participate in this study.

## Author contributions

Conception and design: WG, BW. Provision of study materials or patients: HJ, FH, YY, XC, MS, CZ, BP. Collection and assembly of data: HJ, FH, YY. Data analysis and interpretation: HJ, FH, YY. HJ, FH, and YY contributed equally to this work. WG and BW contributed equally to this work as corresponding authors. All authors contributed to the article and approved the submitted version.

## Supplementary material

Supplementary Figure 1
**(A)** Kaplan–Meier estimates of recurrence-free survival (RFS) for postoperative therapy. **(B)** Kaplan–Meier estimates of RFS for postoperative ctDNA in targeted combined chemotherapy group. (**C)** Kaplan–Meier estimates of RFS for postoperative ctDNA in chemotherapy group.
