# Supplementary figures and images for: Postoperative circulating tumor DNA testing based on tumor naïve strategy after liver metastasis surgery in colorectal cancer patients
Source: Front Oncol. 2023 Apr 28;13:1153685. doi: 10.3389/fonc.2023.1153685 (PMC10198283; doi:10.3389/fonc.2023.1153685)

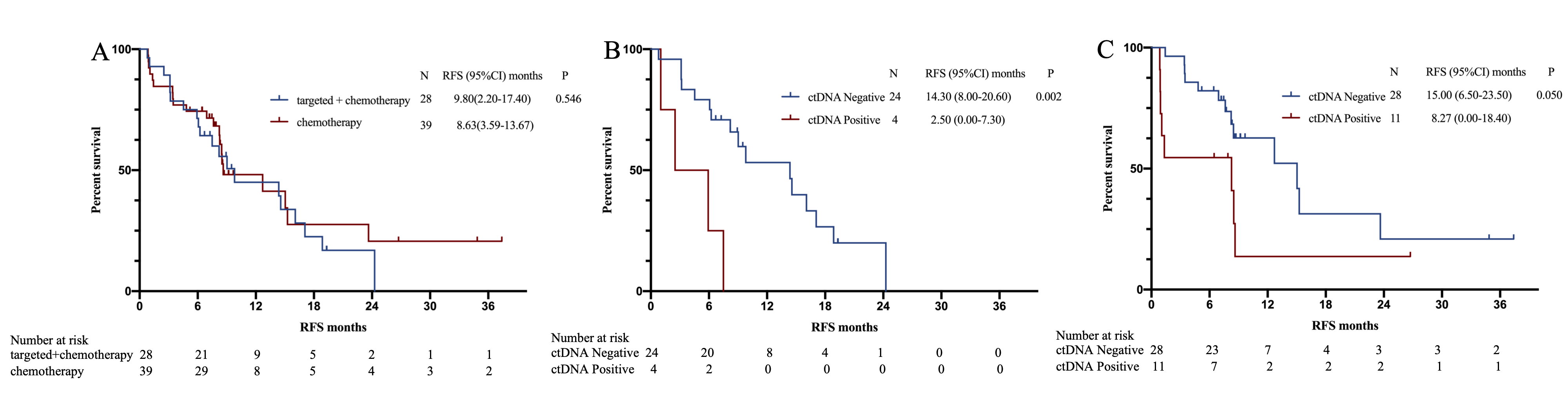

Supplement: Supplementary Figure 1 — (A) Kaplan–Meier estimates of recurrence-free survival (RFS) for postoperative therapy. (B) Kaplan–Meier estimates of RFS for postoperative ctDNA in targeted combined chemotherapy group. (C) Kaplan–Meier estimates of RFS for postoperative ctDNA in chemotherapy group. [file Image_1.tiff]
